# Physiology

**Published:** 1860-03

**Authors:** S. Weir Mitchell

**Affiliations:** Lecturer on Physiology in the Phila. Med. Association


### PHYSIOLOGY.

BY S. WEIR MITCHELL, M.D.,

LECTURER ON PHYSIOLOGY IN THE PHILA. MED. ASSOCIATION.

### I.—*Notice of Further Researches on the Coagulation of the Blood.* By Joseph Lister, Esq., F.R.C.S., Eng. and Edin., etc.

At a meeting of the Medico-Chirurgical Society of Edinburgh, Mr. Lister communicated his views as follows: I may remind the fellows of this Society that, in a paper which I had the honor to read before them the session before last, I brought forward facts which seemed to prove that the ammonia theory does not apply to blood within the vessels of a living animal. That theory asserts that the fluidity of the blood depends upon the presence of a certain amount of free ammonia holding the fibrin in solution, and that coagulation is a necessary result of the escape of the volatile alkali. But it was shown, in the paper referred to, that the blood, in man and other mammalia, though coagulating soon after death in the heart and great venous trunks, remains fluid for days in vessels of smaller size, and this under circumstances affording free opportunity for the escape of ammonia; and, on the other hand, that when a portion of a vessel, either in an amputated limb or in a living animal, is treated in a manner calculated to destroy its vital properties, the blood coagulates in the injured part, but retains its fluidity elsewhere, although there is no greater opportunity for the escape of ammonia in the one case than in the other.

A striking instance of the difference between the natural receptacles of the blood and ordinary matter, in their relations to the vital fluid, happened to come under my notice this morning, in an arm which I amputated at the shoulder-joint last evening, on account of injury inflicted by machinery. On examining the limb, which had lain undisturbed since the operation, I saw that the axillary vein, which was patulous at the part where it had been divided by the knife, contained some blood at a distance of about half an inch from the open orifice; and having squeezed out a few drops, found that it was perfectly fluid, but yielded threads of fibrin when the point of a needle was drawn through it some minutes after emission. The blood had been for twelve hours freely exposed to the air, but being situated in an unwounded part of a blood-vessel, had remained free from coagulation.

I demonstrated also another important principle, viz., that ordinary solid matter, unlike atmospheric air, induces coagulation of blood in its vicinity—when introduced within the living vessels. Having inserted a piece of clean silver wire for a considerable distance into one of the veins of an amputated sheep's foot, I slit up the vessel after a short time had elapsed, when I exhibited a coagulum extending along the whole length of the foreign body; whereas a mere wound of the vein failed to induce a clot, except immediately at the spot where the injury had been inflicted. It was obvious that the introduction of the wire could not affect the amount of ammonia in the blood, and from this and many other facts to which I need not here allude, I was led to the opinion that, as regards what takes place within the living body, the ammonia theory might practically be left entirely out of consideration.

What I have to show now will, I think, prove that, even for blood outside of the body, the ammonia theory, whatever degree of truth it may contain, is very far indeed from representing the whole truth. One of the most remarkable circumstances connected with blood that has been removed from the body is, that it refuses to coagulate below a temperature of 40° F., or thereabout. This is explained by Dr. Richardson, on the hypothesis that the low temperature prevents the evolution of ammonia, while the rapidity with which coagulation takes place at high temperatures seems to him satisfactorily accounted for by the increased volatility exhibited by the ammonia under such circumstances. I was myself disposed at first to accept this interpretation, but subsequent reflection led me to think that, to say the least, it required confirmation. It occurred to me that if it were true that the fluidity of the blood below 40° F. was due to free ammonia retained in it, coagulation would take place immediately, in spite of the cold, if the alkali were neutralized by the addition of acid; provided the fibrin were not impaired in its coagulating property by the reagent employed. In order to ascertain whether this result would really follow, I poured blood freshly shed from a sheep into vessels surrounded by ice-cold water, and by this means succeeded in keeping some portions of it fluid for a considerable time, and found that it continued liquid notwithstanding the addition of diluted acetic acid in what I supposed must be sufficient quantity to overcome the feeble alkalinity of the blood, while the acidulated specimen retained the property of coagulating very rapidly when raised in temperature. But on attempting to discover whether this blood was really acid in reaction, I found that its red color entirely vitiated the indications of both litmus and turmeric; and even the serum obtained after contradiction of the clot was too much tinged to admit of the satisfactory application of the test paper. Being thus baffled in my experiments with the sheep, I had recourse to the horse, in which the red corpuscles subside with peculiar rapidity in the plasma, giving rise to the buffy coat well known to occur in the blood of that animal in the state of health, so that the opportunity would be presented of obtaining liquor sanguinis free from red corpuscles, to which the tests could be applied without risk of fallacy. Accordingly, yesterday afternoon, a horse having been placed at my disposal, by my friend Mr. Gambee, of the New Veterinary College, I tied into the right jugular vein one end of a piece of vulcanized india-rubber tube, four yards in length, the greater part of which was coiled up in a freezing mixture, and some of the blood, having been allowed to remain for a while in the tube, was shed into vessels standing in ice-cold water. Its temperature on first escaping into the air was 39½° F., and having been since kept in the cold, it is still only partially coagulated at the present time, (twenty-nine hours after it was shed.) At first, however, it appeared as if we were likely to fail, the blood of this horse being a rare exception to the general rule, in exhibiting for a long time no appearance of the "sizy" layer. But, after it had stood for about two hours, I succeeded in removing from the surface, by means of a glass tube, a sufficient amount of liquor sanguinis for the performance of an experiment, taking care that the glass into which it was shed and the tube were both near the freezing point. To half a drachm of this plasma I now added one minim and a half of moderately dilute acetic acid, which had the effect of rendering it distinctly acid, as indicated by its communicating a red tint to litmus and restoring the color of turmeric paper which had been reddened by dipping it in the portion of the liquor sanguinis which had not been acidulated. I kept the specimen in ice-cold water till this evening. For a long time it remained perfectly fluid, except the formation of little, soft coagulum at the surface, just as in the unacidulated blood; but a few drops placed in a watch-glass and brought into a warmer atmosphere, coagulated in about the same time as the blood that first flowed from the tube, a soft clot forming in about a quarter of an hour. Even at the expiration of twenty-four hours, a portion of what remained in the cold was still fluid, though faintly acid, but set into a pretty firm clot on being removed into a warmer situation.

[Mr. Lister now proceeded to perform a similar experiment before the Society. A glass containing some liquor sanguinis of the horse's blood, shed twenty-nine hours before, was taken out of the mixture of ice and water in which it stood, and the contents were seen to be still to a considerable extent fluid, although acidulated with acetic acid two hours previously. A portion of the liquid was poured into a watch-glass, and, having been shown to be acid by litmus-paper, was set aside to coagulate, and about a quarter of an hour later was exhibited as a soft clot. Mr. L. then continued.]

From these facts it is obvious that the ammonia theory utterly fails to explain the influence of temperature on coagulation. The circumstance that the liquor sanguinis was acid in this experiment, is clear proof that it contained no free ammonia whatever; yet the acidulated plasma was affected by cold and heat just like ordinary blood. It remained fluid near the freezing point, although the ammonia it originally contained must have entered into combination and lost its reputed power of dissolving the fibrin, and it coagulated when warmed, though the ammonia, fixed by the acid, must have been incapable of evolution. If the author of the ammonia theory were asked to explain why this horse's blood took a quarter of an hour to coagulate, he would no doubt reply that it must have contained a large amount of ammonia, requiring all this time to escape. But we have seen that the acid liquor sanguinis, though possessing no free ammonia at all, took as long to clot. There can, therefore, I think, be little question, but that the slowness of coagulation in the horse, compared with the rapidity of the process in the sheep, and the variations met with in the period in the human species, depend not on the amount of ammonia present in the blood, but on differences in its other constituents, and, speaking generally, that the theory which attributes the coagulation of the blood to the escape of ammonia is fallacious.—(*Ed. Med. Monthly*, December, 1859.)

II.—*Experiments as to the Production of Epilepsy in Guinea-pigs.* By M. BROWN-SÉQUARD.

One of the most interesting facts discovered by M. Brown-Séquard is that relative to the production in mammalia, and particularly in Guinea-pigs, of an epileptiform condition, the result of various injuries inflicted upon the spinal cord. M. Brown-Séquard has often repeated these experiments with the same results, and has shown them to different learned societies. At the last meeting of the Society of Biology, (29th October, 1859,) he announced that for some years past he has had an opportunity of observing a considerable number of young Guinea-pigs born of parents which he had rendered epileptic. But in some of these young animals he has recognized a distinct epileptiform affection, with paroxysms well characterized, yet differing somewhat from that observed in the parents. In the case of the parents, there are not only spontaneous paroxysms, but, in addition, a fit can be brought on at any time by pinching the skin of the face. In those Guinea-pigs which appear to inherit the convulsive affection from their parents, the occurrence of paroxysms cannot in this way be determined; neither are the symptoms of the fit in the young animals quite the same; at the commencement of the attack the animal is seized with trembling, then it falls upon its side and agitates its limbs convulsively.

The Guinea-pigs so affected, which M. Brown-Séquard has in his possession at present, are of two kinds, which are nearly equal in number—one set being the offspring of a mother rendered epileptic by an injury of the spinal cord, the others of a father placed in the same condition.

It may further be observed, that parents rendered epileptic by injury of the spinal column give birth to young ones, none of which may suffer from the affection, or of which some may be free from, while others may suffer from convulsive attacks. M. Brown-Séquard has had under observation a very large number of Guinea-pigs, and although he does not deny the possibility of the fact, he has never seen one of them suffer from a similar spasmodic affection, unless it had previously received an injury of the spinal cord, or were the offspring of a parent rendered epileptic by such proceeding.

These observations of M. Brown-Séquard are of great value; for they add a new point of resemblance to those which already assimilate epilepsy in man to the convulsive affection produced in mammalia by injuries of the spinal cord. This new feature is hereditary transmission. The greater the analogy between the two diseases, the more will the study of epilepsy in animals help the solution of the difficult problems which still surround the history of that disease in man.

M. Brown-Séquard has also pointed out that these facts have another kind of interest. It is well known that traumatic lesions are scarcely ever handed down by hereditary transmission, or at least that this transmission is very rare. Now the above observations might be adduced as examples demonstrative of the possibility of this transmission, but this would be erroneous. In fact, in the offspring of epileptic Guinea-pigs, the spinal cord, examined either with the naked eye or with the microscope, appears perfectly healthy. The local lesion then is not transmitted; it is the general alteration or disposition of the nervous system determined by the lesion, and which is profoundly impressed upon both parents or upon one of them, which is inherited by the offspring. We ought rather, then, to class these examples with cases of hereditary transmission of a diathetic affection produced in an individual, male or female, by some well determined cause.—(*Gazette Hebdomadaire; Edinb. Med. Journal*, December, 1859.)

III.—*On the Cause of Death in Animals subjected to High Temperatures.*

M. Claude Bernard states that warm-blooded vertebrate animals, inclosed in a dry and heated atmosphere, live for a certain time without any appreciable effect being indicated, except accelerated respiration and circulation, death occurring suddenly after the lapse of some time, which varies with the elevation of temperature, the size of the animal, its age, etc. The cause of death in these cases, he believes to be attributable solely to the elevation of temperature of the blood, independently of other circumstances. In the mammifera and birds, the temperature may be raised from four to five degrees C. above the normal condition, the animals infallibly perishing whenever this limit is exceeded, and at the moment of death the heart being found rigid along with the other muscles of the body. The interesting conclusion drawn from his experiments is, that a purely physical condition of the blood—mere increase of its temperature—may be a cause of death. And another point also interesting in connection with this circumstance is, that the amount of increase in temperature sufficient to produce this effect is well marked and invariable, being from four to five degrees C. above the normal standard in warm-blooded animals—that is, between forty-five and forty-six degrees C. in mammifers, and fifty-one and fifty-two degrees C. in birds. This M. Bernard explains as arising from the fact that such increase of temperature is incompatible with the exercise of muscular contractility, and consequently leads to arrestment of the heart's action. M. Kühne has shown that in such cases the rigidity is due to the coagulation of a certain matter contained in muscular tissue.—(*Gaz. Medicale*, July, 1859; *Edinb. Med. Journal*, December, 1859.)

IV.—*Presence of Urea in Chyle and Lymph.* By M. AD. WURTZ.

Two years ago M. Wurtz, finding at Alfort a carnivorous bull, in which a fistula of the thoracic duct had been established, it occurred to him to search for urea in the chyle of this animal. To this inquiry he was induced by the consideration that urea ought to originate, not in the capillary blood-vessels, as has been sometimes pretended, but in the intimate structure of all the tissues at every part where materials which have become unfit for life require to be removed by the respiratory combustion. If this be the case, we ought to detect urea not only in the blood, where its presence has been ascertained for a long time past, but also in lymph, and consequently in the chyle of the thoracic duct. It appears, indeed, natural that the lymphatics should contribute their share toward the absorption of the materials proceeding from the metamorphoses of the tissues in which the radicles of these vessels are distributed. The chyle of the bull referred to was accordingly found very rich in urea. M. Wurtz coagulated, at a warm temperature, about 600 grammes of the chyle, evaporated the filtered liquor, treated it with absolute alcohol, evaporated, and then exhausted the alcoholic extract by means of ether; the latter deposited perfectly colorless crystals of urea, which was partially converted into nitrate. This induced M. Wurtz to extend his researches to lymph. Having procured, through the kindness of M. Colin, the lymph of the dog, the cow, the bull, and the horse, he ascertained in them the presence of urea. It further appeared of interest to compare the quantities of urea contained in the blood, chyle, and lymph of the same animal. For this purpose it was necessary to undertake certain quantitative researches, which were accomplished by means of a process which is too long to explain, but which is essentially founded on a combination of the methods which MM. Liebig and Bunsen have proposed for the determination of urea. The following table presents the numerical results obtained in these researches:—

| NAME OF ANIMAL. | DIET. | Quality of Urea contained in 1000 Grammes of | | |
|---|---|---|---|---|
| | | Blood. | Chyle. | Lymph. |
| Dog. | Flesh. | 0·089 | ...... | 0·158 |
| Do. | Do. | ...... | 0·183 | ...... |
| Cow. | Dried lucern. | 0·192 | 0·192 | 0·193 |
| Bull. | Lucern and rape-cake. | ...... | 0·189 | 0·213 |
| Do. | Oil-cake before rumination. | ...... | ...... | 0·215 |
| | | Arterial. | | |
| Ram. | Ordinary diet—rumination suspended. | 0·248 | 0·280 | ...... |
| Sheep. | Do. do. do. do. | ...... | 0·071 | ...... |
| Horse. | Do. do. do. do. | ...... | ...... | { 0·126<br>0·112 |

M. Wurtz adds, that having had occasion to analyze a certain quantity of chyle properly so called, collected along the course of the mesenteric chyliferous vessels, after the ganglions, he ascertained there also the presence of a small quantity of urea. This is, no doubt, derived from the changes of tissue which are effected in the walls of the intestine itself.—(*Comptes Rendus de l'Acad. des Sciences*, July, 1859; *Edinburgh Medical Journal*, December, 1859.)

